# Rethinking Communication Barriers: Educational Attainment in Cervical Cancer Screening Among American Sign Language Users

**DOI:** 10.3390/ejihpe16070097

**Published:** 2026-07-09

**Authors:** Hiruni Hewapathiranage-Mayadunne, Erika Bergeron, Poorna Kushalnagar

**Affiliations:** Center of Deaf Health Excellence, Gallaudet University, Washington, DC 20002, USA; hiruni.hewapathirana-mayadunne@gallaudet.edu (H.H.-M.); erika.bergeron@gallaudet.edu (E.B.)

**Keywords:** cervical cancer screening, deaf, educational attainment, English proficiency, screening adherence, healthcare disparities

## Abstract

Deaf, deafblind, and hard-of-hearing (DDBHH) American Sign Language (ASL) users assigned female at birth experience disparities in cervical cancer screening. There is a need to further clarify the roles of education status and English proficiency in adherence to the U.S. Preventive Services Task Force (USPSTF) screening guidelines. Using the NCI Health Information National Trends Survey in ASL, we surveyed participants; those who reported being female and within the USPSTF age-eligible range (21–65) were included in analyses. Associations were determined using Pearson Chi-squared tests. A two-sided *p* ≤ 0.05 was considered significant. Six hundred and forty-three participants, recruited between July 2023 and October 2025, answered questions in ASL and English, with 65% self-reporting screening adherence. Chi-square results indicated that more years of education were significantly associated with screening adherence [X2 (2) = 9.72, *p* = 0.008]. Consistent with the results, English proficiency was not a significant predictor of screening adherence [Fisher’s exact *p* = 0.267]. Those who self-report as HS graduates and/or have left college demonstrate lower adherence as well. These findings suggest that educational attainment may be an important factor associated with screening adherence among DDBHH ASL users and warrants further investigation in efforts to improve adherence toward Healthy People 2030 targets.

## 1. Introduction

Cervical cancer is the fourth most common cancer and cause of cancer death in women worldwide, despite being largely preventable through screening. In the United States, widespread Pap smear screening programs have successfully reduced cervical cancer mortality by identifying precancerous changes before they progress. Screening adherence, however, varies significantly across populations. Among (DDBHH) individuals assigned female at birth who use American Sign Language (ASL), adherence rates reflect disparities found in the general population, with certain subgroups at higher risk for non-adherence than others. Healthcare providers often assume that communication barriers are the primary obstacle and that providing ASL interpreters adequately addresses these challenges (Perrodin-Njoku et al., 2022). Language differences alone do not determine healthcare outcomes. Existing evidence shows that dominant-language proficiency predicts health literacy when health information is available only in that language (Jacobson et al., 2016); far less is known about populations for whom health information is accessible in their primary language. Such a basis for care is far more common among populations whose primary language is accessible to them, and we cannot assume the same for those dominant in minority languages. Research consistently demonstrates that educational attainment is a significant predictor of health information seeking among U.S. adults, and that health information seeking is, in turn, positively associated with cancer-related outcomes, including screening adherence (Vazquez et al., 2024; Wigfall & Friedman, 2016). Educational attainment has been associated with greater health information-seeking behaviors, health literacy, and engagement with preventive healthcare services, factors that may facilitate adherence to recommended screening practices. For DDBHH ASL users assigned female at birth, this has important implications for health outcomes. Past works established that language use and education were each associated with screening when comparing DDBHH and hearing samples. The present study instead examines variation within the bilingual DDBHH population, whose members use ASL as their primary language alongside varying levels of English proficiency. Within this population, English proficiency did not predict screening adherence, leaving educational attainment, a social determinant of health, as the more informative factor. This distinction matters clinically: providers may assume that patients who are not fluent in English also have limited literacy, whereas attending to a patient’s educational attainment offers a less biased basis for identifying who may benefit from additional screening support. This paper examines how educational attainment, rather than English proficiency alone, affects adherence to cervical cancer screening guidelines, and argues that healthcare providers must assess these factors rather than stopping their evaluation at language access.

Educational attainment among DDBHH individuals ranges widely, from those who attended residential schools for the deaf with strong bilingual ASL–English instruction to those who attended schools with limited support that restricted students’ access to classroom information (e.g., interpretation and captioning). This educational diversity, combined with varying levels of English proficiency, creates heterogeneity in health outcomes that mirrors patterns seen in other linguistic communities. Yet research on DDBHH populations frequently aggregates subgroups, which can conceal disparities in screening uptake within the broader DDBHH community (Perrodin-Njoku et al., 2022). Research on cervical cancer screening among DDBHH women reflects this tendency to overlook within-group variation. Kushalnagar and colleagues found that American women who use ASL had a cervical cancer screening adherence rate of 78% compared to 85% among American women who use English (Kushalnagar et al., 2019). While this difference is statistically significant, the study positioned language use as the primary variable without examining other factors that might explain variation in screening adherence within the DDBHH population itself, such as educational attainment or English proficiency. This pattern is consistent with findings from earlier research on language and healthcare disparities.

In contrast, Amboree and colleagues examined cervical cancer screening among Hispanic women and found that disaggregating by factors beyond language revealed important within-group differences (Amboree et al., 2023). Spanish-speaking women with county healthcare coverage had higher screening rates than those with private insurance. This suggests that structural factors such as insurance type and access to care coordination matter independently of language preference alone. These studies show that treating a linguistically distinct community as a homogenous group defined solely by language use obscures the role. Research on immigrant populations provides a useful framework for how education and primary-language proficiency influence healthcare navigation. Cudjoe et al. (2021) found that African immigrant women adhered to Pap screening guidelines at rates comparable to English speakers when given language-appropriate resources. Building on this, Chu et al. (2022) found that educational attainment and fluency in one’s primary language were associated with more effective healthcare engagement among multilingual populations, whereas English proficiency alone was less consistently associated with engagement. Xie et al. (2023) found that limited English proficiency was independently associated with lower screening adherence among Asian American adults. These findings suggest that healthcare systems often privilege English-language communication, creating barriers to screening that may be reduced through structural supports, higher educational attainment, and access to language-concordant resources.

Applying this framework to DDBHH populations reveals an important research gap. If education and proficiency in one’s primary language predict screening adherence across immigrant populations, these same factors likely explain variation in adherence rates among DDBHH ASL users assigned female at birth. Research in DDBHH populations has predominantly focused on communication barriers and interpreter provision, with limited attention to whether educational attainment predicts screening adherence, especially when screening recommendations are communicated in patients’ primary language (Wang et al., 2026; Kushalnagar et al., 2018). This paper addresses this gap by examining how educational attainment and English proficiency predict screening adherence among DDBHH ASL users assigned female at birth.

## 2. Materials and Methods

Measures: Survey items assessed demographics, cancer screening history, and English use, aligning with USPSTF cancer screening guidelines [see Kushalnagar et al. (2017) for methodology]. All measures were self-reported. The participants were asked the following:

- “What was your sex assigned at birth?” Response options included: female; male; intersex.
- “How long ago did you have your most recent Pap test to check for cervical cancer?” Response options included: a year ago or less; more than 1, up to 2 years ago; more than 2, up to 3 years ago; more than 3, up to 5 years ago; more than 5 years ago; I have never had a Pap test. This item captured the timing of the most recent Pap test only and did not record whether concurrent HPV testing was performed. Because we could not distinguish respondents who had undergone HPV co-testing, we applied the cytology-alone interval from the USPSTF cervical cancer screening recommendation in effect during the data collection period and defined guideline-concordant screening as having completed a Pap test within the past three years. Respondents reporting a Pap test within the past three years were classified as adherent; all others were classified as non-adherent.
- “What is the highest grade or level of schooling you completed?” Response options included: less than 8 years; 8 through 11 years; 12 years or completed HS; post high school training; some college; college graduate; postgraduate.
- “How well do you use English?” Response options included: very well; well; not well; not at all.

Data Collection and Eligibility: An ASL version of the National Cancer Institute’s (NCI) Health Information National Trends Survey (HINTS) was used to gather data from age-eligible adults who use ASL between July 2023 and October 2025 (Kushalnagar et al., 2017). Eligible participants were individuals who (1) self-identified as DDBHH; (2) used ASL as their primary language; (3) were between 21 and 80 years of age; and (4) were residents of the United States. For this study, we included data from adult participants assigned female at birth to assess the USPSTF guidelines’ age-appropriate adherence to cervical cancer screening. Respondents reporting a Pap test within the past three years were classified as adherent; all others were classified as non-adherent.

Consenting process: Prior to survey participation, participants watched an ASL video accompanied by English text that explained the study’s purpose, procedures, potential risks, and benefits. Before providing signed informed consent, prospective participants were given the opportunity to ask questions and review inclusion/exclusion criteria with research staff in ASL.

Procedure: Recruitment was conducted by the Center of Deaf Health Excellence (CDHE) across three venue types: local community events, national DDBHH-centered events, and catchment-area events held with community partners. All recruitment abided by a standardized protocol that included participant identification, eligibility screening, consent verification, survey completion, and compensation distribution. Participants first completed name verification to prevent duplicate enrollment, followed by eligibility screening confirming age, ASL use, and U.S. residency. The bilingual survey took approximately 15 min to complete, with research staff available for technology or clarification of survey items. After survey completion, a remote researcher confirmed survey completion and processed gift card compensations.

Ethical Considerations and Compensation: This study was approved by the Institutional Review Board at Gallaudet University. Participants received a $10 gift card for survey completion. To ensure confidentiality, participants’ identifying information was stored separately from their survey responses and linked only via unique identifiers.

Statistical Analysis: Descriptive statistics were calculated for all characteristics—overall and by screening status (adherent/non-adherent). Between-group differences were assessed using Fisher’s exact tests. We obtained Odds Ratios (ORs), 95% confidence intervals (CIs) and the log-likelihood ratios from separate logistic regressions that included (1) only English use; (2) only education; (3) English use and education, adjusting for age; and (4) an interaction of age with education, race, and marital status. The interaction of age with education was included as we believed that the relationship of English with cervical cancer screening would vary by education within each age group. A two-sided *p* ≤ 0.05 was considered significant. All measures were self-reported; educational attainment was collapsed into HS degree or less, some college, and college; English use was dichotomized as well/not well; and cervical cancer screening adherence was defined as a Pap test within the past three years.

Associations between Pap smear adherence (screened vs. not screened), self-reported English proficiency (well vs. not well), and education status (HS degree or less, some college, and college) were assessed using Pearson Chi-squared tests. A two-sided *p* ≤ 0.05 was considered significant. For this study, eligible participants assigned female at birth who are 21 to 65 years old and who provided consent were included in the analysis for cervical cancer screening adherence. Data included demographics, cancer screening history, and health insurance.

## 3. Results

Six hundred and forty-three DDBHH participants assigned female at birth, aged 21–65 (M = 49, SD = 11), completed a cancer screening survey in ASL between July 2023 and October 2025. Within this group, 65% were adherent to screening guidelines. Education was the only variable significantly associated with adherence (χ^2^ = 9.72, df = 2, *p* = 0.008): adherence was highest among college graduates (68.3%), followed by those with some college (56.3%) and a high school degree (51.2%). English use was not significantly associated with adherence (Fisher’s exact *p* = 0.267). Similarly, race/ethnicity (χ^2^ = 2.47, df = 4, *p* = 0.653) and marital status (χ^2^ = 2.74, df = 5, *p* = 0.743) showed no significant differences (Table 1). Age group approached but did not reach significance (χ^2^ = 5.85, df = 2, *p* = 0.052), with a trend toward lower adherence among those aged 50–65 (60.4% of the non-adherent group).

Table 2 presents results from three logistic regression models predicting cervical screening adherence. In Model 1, English use alone was not significantly associated with adherence (OR = 1.34, 95% CI: 0.84–2.15, *p* = 0.224; LR χ^2^ = 1.46, df = 1, *p* = 0.225). In Model 2, education alone was a significant predictor of adherence (LR χ^2^ = 9.49, df = 2, *p* = 0.009); compared to college graduates, those with some college (OR = 0.60, 95% CI: 0.40–0.90, *p* = 0.014) and a high school degree (OR = 0.49, 95% CI: 0.26–0.93, *p* = 0.028) had lower odds of adherence.

The full adjusted model (Model 3) did not reach overall significance (LR χ^2^ = 22.03, df = 15, *p* = 0.107); education effects were attenuated following adjustment for age and age × education interactions, with wide confidence intervals reflecting limited statistical power. Hispanic ethnicity was the only individually significant predictor (OR = 1.66, 95% CI: 1.04–2.64, *p* = 0.033), while English use remained non-significant (OR = 1.44, 95% CI: 0.89–2.34, *p* = 0.140). Educational attainment was the strongest bivariate predictor of adherence; however, this effect was not maintained after covariate adjustment.

## 4. Discussion

Among 643 DDBHH ASL users assigned female at birth in our study, many are not guideline-adherent. The factors driving this are not well understood and remain underexamined in the existing literature. National estimates (e.g., NHIS & ODPHP, 2021) suggest screening may be lower in this population, but the data do not permit a direct comparison. English use was not significantly associated with cervical cancer screening adherence in either bivariate or adjusted analyses. This finding runs counter to prior research suggesting that English proficiency is a meaningful predictor of Pap smear adherence in the general population that uses primarily English. This null finding likely reflects underlying structural factors. In a DDBHH sample, systemic communication barriers are nearly universal regardless of English proficiency level. English use does not meaningfully differentiate screening behavior within this population. DDBHH patients arrive at clinical encounters with fewer opportunities to build health literacy, not due to individual limitation, but because health information systems were not designed with them in mind. Providers should account for this structural reality proactively by meeting patients where they are at with appropriate resources and approaches (McKee et al., 2015).

It is also shown that DDBHH ASL users assigned female at birth experience lower rates of health and cancer knowledge. According to a study conducted by Spellun and colleagues, 58% of DDBHH participants compared to 84% of hearing participants reported knowing about HPV, indicating a significant disparity (Spellun et al., 2019). As the DDBHH population faces social barriers, they experience more communication difficulties. Spellun and colleagues also found that DDBHH young adults are more likely to have limited knowledge of their own family medical history, a result of limited access to incidental health conversations in family settings where ASL is not used. Additionally, Bergeron and colleagues found that the DDBHH population encounters additional barriers beyond communication, including a lack of insurance access, inaccessible patient portals, and providers’ lack of understanding of how to interact with and provide accessibility for DDBHH patients (Bergeron et al., 2024). In the U.S., most available health information is primarily in English and is not designed to meet the communication needs of DDBHH ASL users. A study by Cudjoe and colleagues found that women highly proficient in English were 50% more likely to adhere to Pap smear testing guidelines than those not proficient in English (Cudjoe et al., 2021). This is a pattern that was not replicated in the present DDBHH sample, further suggesting that the mechanisms linking spoken language environment to screening behavior may differ for this population.

DDBHH Americans who primarily use ASL are systematically excluded from health information by a healthcare system designed for those who use spoken English. In a community health needs assessment of DDBHH ASL users in Florida, James et al. (2022) found that 37.2% of participants who visited a medical facility for mental health services within the past year were denied interpretation services. Although that assessment did not focus on the preventive-care settings where cervical cancer screening occurs, this barrier may be present throughout the cervical cancer screening process for individuals who use ASL.

Jacobson et al. (2016) discuss Cummins’ language interdependence principle, which posits that proficiency in a first language (L1) supports proficiency in a second language (L2). This framework highlights the need for future assessments of health literacy to consider both language proficiency and educational attainment. Evaluating a patient’s ability to communicate in a non-primary language without accounting for their L1 proficiency and educational background may conceal their true level of health literacy. As a result, physicians may be unable to accurately assess a patient’s understanding of health information and care instructions. This pattern may extend across the cancer screening continuum, where language proficiency and educational attainment, aligned with language-concordant materials, can influence patients’ understanding of screening recommendations and their adherence. Healthcare systems need to be redesigned to meet DDBHH patients where they are at. The most effective response to these structural barriers is not to make DDBHH patients more accessible to healthcare. It is to make healthcare more accessible to DDBHH patients. Language-concordant, ASL-fluent community health navigators (CHNs) represent one promising avenue for delivering targeted and patient-centered screening support. A randomized controlled trial currently underway is examining whether ASL-fluent CHNs delivering education and navigation via videoconferencing can improve cancer screening adherence among DDBHH adults (Kushalnagar et al., 2025).

In bivariate analysis, educational attainment was associated with adherence; however, this association was not maintained after adjusting for other covariates in the regression analysis. Age group and race/ethnicity were not significantly associated with adherence in bivariate analysis and did not emerge as significant predictors in adjusted models. Descriptively, adherence rates varied by race/ethnicity, though differences did not reach statistical significance and should be interpreted as descriptive rather than indicative of differential risk within this sample. The sample was recruited by convenience and was highly educated and largely self-reported English-proficient participants, with only 83 respondents (13.0%) reporting limited English use; this constrains generalizability to the broader DDBHH population and limits power to detect associations within smaller subgroups, particularly respondents reporting limited English use. The graded bivariate association between education and adherence across all three education levels suggests that the overall pattern is unlikely to be an artifact of sample composition, and confirmation in a larger, more diverse sample is warranted.

These findings underscore the importance of providers approaching each DDBHH patient as an individual, drawing on cultural humility and patient-centered care rather than defaulting to communication mode or English proficiency as a proxy for health literacy or adherence risk. Within this DDBHH sample, educational attainment was associated with screening adherence in bivariate analyses and may help identify patients who could benefit from additional support; however, this association was attenuated after adjustment for demographic covariates and should be interpreted cautiously.

Proactive engagement from healthcare providers is necessary for DDBHH patients to engage in discussions and understand content during clinical encounters. Providers should consider patients’ educational experiences as part of a broader assessment of health literacy and screening needs, rather than relying solely on English proficiency as an indicator of readiness to engage with screening recommendations. As the present findings demonstrate, English proficiency alone did not predict cervical screening adherence in this deaf, deafblind, and hard-of-hearing sample; even patients who communicate well in English may face educational or informational gaps that affect their understanding of screening guidelines.

Relying on passive, language-only accommodation solutions like providing a pamphlet in English or language interpretation is insufficient when the underlying barrier is not fluency but access to substantive, education-informed health information delivered in a language-concordant format. In other words, that approach invites more confusion and dejection, further lowering the adherence rate. Research in analogous underserved populations demonstrates that community-tailored narrative approaches can increase cervical cancer screening uptake, which suggests that intervention design is as important as access (Ochoa et al., 2020). For DDBHH ASL users assigned female at birth specifically, proactive and individualized outreach delivered by ASL-fluent community health navigators represents a more effective foundation for supporting guideline-concordant cervical cancer screening than passive accommodation alone (Bergeron et al., 2024). Language-concordant, ASL-fluent community health navigators are well positioned to bridge this gap, providing both individualized and education-informed outreach and support. This offers a scalable model for improving guideline-concordant cervical cancer screening adherence among DDBHH ASL users assigned female at birth (Kushalnagar et al., 2025).

## 5. Conclusions

Among 643 DDBHH ASL users assigned female at birth, 65% reported adherence to guideline-concordant cervical cancer screening. Although direct comparison is limited by differences in sampling and survey methods, national estimates suggest adherence in this population may fall below that of the general population. Educational attainment was the strongest predictor in bivariate analyses, though this association was attenuated after adjustment for covariates. English use was not significantly associated with screening adherence in any model.

These findings suggest that English proficiency alone is not an adequate proxy for screening risk in the DDBHH population. Educational attainment may serve as a useful indicator for identifying patients who could benefit from additional support in understanding and completing recommended screening.

Healthcare providers should adopt person-centered, responsive approaches that account for individual educational experiences rather than relying on language-based assumptions. Language-concordant community health navigators represent a promising strategy for improving screening access and adherence among DDBHH populations.

## Figures and Tables

**Table 1:** Descriptive statistics by cervical screening adherence (N = 643).

Variable	Overall (N = 643)	Adherent (n = 418)	Non-Adherent (n = 225)	χ^2^ (df)	*p*-Value
Age, mean (SD)	48.9 (11.3)	48.4 (11.2)	49.9 (11.7)	5.85 (2)	0.052
Age group, n (%)					
21–39	148 (23.0%)	97 (23.2%)	51 (22.7%)		
40–49	141 (21.9%)	103 (24.6%)	38 (16.9%)		
50–65	354 (55.1%)	218 (52.2%)	136 (60.4%)		
Self-reported English use, n (%)				Fisher’s Exact	0.267
Well (very well/well)	556 (87.2%)	365 (88.4%)	189 (84.8%)		
Not well (not well/not at all)	83 (13.0%)	49 (11.9%)	34 (15.2%)		
Missing	6	4	2		
Education, n (%)				**9.72 (2)**	**0.008**
College graduate	483 (75.1%)	330 (78.9%)	153 (68.0%)		
Some college	119 (18.5%)	67 (16.0%)	52 (23.1%)		
HS degree	41 (6.4%)	21 (5.0%)	20 (8.9%)		
Race/ethnicity, n (%)				2.74 (5)	0.743
Married	275 (42.8%)	180 (43.1%)	95 (42.2%)		
Living with a partner	59 (9.2%)	33 (7.9%)	26 (11.6%)		
Divorced	105 (16.3%)	69 (16.5%)	36 (16.0%)		
Widowed	31 (4.8%)	20 (4.8%)	11 (4.9%)		
Separated	8 (1.2%)	6 (1.4%)	2 (0.9%)		
Never married	165 (25.7%)	110 (26.3%)	55 (24.4%)		

Note: Percentages calculated among non-missing observations. Fisher’s exact test used for English use (2 × 2 table); Pearson’s χ^2^ test used for all other categorical variables. Bold *p*-value indicates significance at α = 0.05.

**Table 2:** Logistic regression models predicting cervical screening adherence.

Variable	Model 1: Self-Reported English Use Only		Model 2: Education Only		Model 3: Full Model	
	OR (95% CI)	*p*	OR (95% CI)	*p*	OR (95% CI)	*p*
Self-Reported English use (ref: Not Well)						
Well (very well/well)	1.34 (0.84, 2.15)	0.224	—	—	1.44 (0.89, 2.34)	0.140
Education (ref: College Graduate)						
Some college	—	—	0.60 (0.40, 0.90)	0.014	0.21 (0.03, 1.50)	0.120
HS degree	—	—	0.49 (0.26, 0.93)	0.028	1.35 (0.02, 115.17)	0.895
Age (continuous)	—	—	—	—	0.99 (0.97, 1.01)	0.265
Age × Education Interactions						
Age × some college	—	—	—	—	1.02 (0.98, 1.06)	0.346
Age × HS degree	—	—	—	—	0.98 (0.90, 1.06)	0.596
Race/Ethnicity (ref: White)						
Hispanic	—	—	—	—	**1.66 (1.04, 2.64)**	**0.033**
African American/Black	—	—	—	—	1.53 (0.85, 2.76)	0.154
Asian	—	—	—	—	1.06 (0.55, 2.04)	0.858
Other	—	—	—	—	0.87 (0.33, 2.25)	0.770
Marital Status (ref:)						
Living with a partner	—	—	—	—	0.66 (0.36, 1.20)	0.171
Divorced	—	—	—	—	0.94 (0.57, 1.53)	0.790
Widowed	—	—	—	—	0.98 (0.44, 2.17)	0.956
Separated	—	—	—	—	1.41 (0.27, 7.25)	0.683
Never married	—	—	—	—	1.05 (0.69, 1.61)	0.813
MODEL FIT STATISTICS						
N	637		643		635	
Log-Likelihood	−411.72		−411.53		−399.34	
Null Log-Likelihood	−412.45		−416.28		−410.35	
Pseudo R^2^ (McFadden)	0.0018		0.0114		0.0268	
LR Test Statistic	1.46		9.49		22.03	
LR Test df	1.00		2.00		15.00	
LR Test *p*-value	0.225		**0.009**		0.107	

Note: OR = Odds Ratio; CI = Confidence Interval. — indicates variable not included in model. Model 1: Unadjusted English use only. Model 2: Unadjusted education only. Model 3: English use and education, adjusted for age, age × education interaction, race/ethnicity, and marital status. Reference categories: not well (English use), college graduate (education), White (race/ethnicity), married (marital status). Bold *p*-values indicate statistical significance at α = 0.05.

## Data Availability

The datasets presented in this article are not readily available because the participant is from a hard-to-reach population that is small and risks being identified. Requests to access the datasets should be directed to poorna.kushalnagar@gallaudet.edu.
